# Characterization of SARS-CoV-2 RNA, Antibodies, and Neutralizing Capacity in Milk Produced by Women with COVID-19

**DOI:** 10.1128/mBio.03192-20

**Published:** 2021-02-09

**Authors:** Ryan M. Pace, Janet E. Williams, Kirsi M. Järvinen, Mandy B. Belfort, Christina D. W. Pace, Kimberly A. Lackey, Alexandra C. Gogel, Phuong Nguyen-Contant, Preshetha Kanagaiah, Theresa Fitzgerald, Rita Ferri, Bridget Young, Casey Rosen-Carole, Nichole Diaz, Courtney L. Meehan, Beatrice Caffé, Mark Y. Sangster, David Topham, Mark A. McGuire, Antti Seppo, Michelle K. McGuire

**Affiliations:** a Margaret Ritchie School of Family and Consumer Sciences, University of Idaho, Moscow, Idaho, USA; b Department of Animal, Veterinary, and Food Sciences, University of Idaho, Moscow, Idaho, USA; c Department of Pediatrics, Division of Allergy and Immunology, University of Rochester School of Medicine and Dentistry, Rochester, New York, USA; d Department of Pediatric Newborn Medicine, Brigham and Women’s Hospital and Harvard Medical School, Boston, Massachusetts, USA; e David H. Smith Center for Vaccine Biology and Immunology, Department of Microbiology and Immunology, University of Rochester Medical Center, Rochester, New York, USA; f Department of Anthropology, Washington State University, Pullman, Washington, USA; University of Pittsburgh School of Medicine

**Keywords:** COVID-19, SARS-CoV-2, antibodies, breastfeeding, breastmilk, human milk, neutralizing capacity

## Abstract

Whether mother-to-infant SARS-CoV-2 transmission can occur during breastfeeding and, if so, whether the benefits of breastfeeding outweigh this risk during maternal COVID-19 illness remain important questions. Using RT-qPCR, we did not detect SARS-CoV-2 RNA in any milk sample (*n *= 37) collected from 18 women following COVID-19 diagnosis. Although we detected evidence of viral RNA on 8 out of 70 breast skin swabs, only one was considered a conclusive positive result. In contrast, 76% of the milk samples collected from women with COVID-19 contained SARS-CoV-2-specific IgA, and 80% had SARS-CoV-2-specific IgG. In addition, 62% of the milk samples were able to neutralize SARS-CoV-2 infectivity *in vitro*, whereas milk samples collected prior to the COVID-19 pandemic were unable to do so. Taken together, our data do not support mother-to-infant transmission of SARS-CoV-2 via milk. Importantly, milk produced by infected mothers is a beneficial source of anti-SARS-CoV-2 IgA and IgG and neutralizes SARS-CoV-2 activity. These results support recommendations to continue breastfeeding during mild-to-moderate maternal COVID-19 illness.

## Abstract (precis)

Results from prior studies assaying human milk for the presence of SARS-CoV-2, the causative virus of COVID-19, have suggested milk may act as a potential vehicle for mother-to-child transmission. Most previous studies are limited because they followed only a few participants, were cross-sectional, and/or failed to report how milk was collected and/or analyzed.

## INTRODUCTION

The global spread of severe acute respiratory coronavirus 2 (SARS-CoV-2), the causative agent of coronavirus disease 2019 (COVID-19), has led to concerns over mother-to-child transmission, including via breastfeeding. Several studies have reported the presence of SARS-CoV-2 RNA in human milk (1–4), whereas others have not (5–9) (see Table S1 in the supplemental material). Most previous studies are limited because they followed only a few participants, were cross-sectional, and/or failed to adequately report how milk was collected and/or analyzed. Thus, some uncertainty remains regarding whether human milk is capable of transmitting SARS-CoV-2 from mother to infant.

10.1128/mBio.03192-20.3 TABLE S1 Studies examining human milk for evidence of SARS-CoV-2 RNA via RT-qPCR. *Buonsenso et al., 2020, and Costa et al., 2020, reported on the same participants and are both included for completeness, but not duplicated in the total counts. **Slaats et al., 2020, reported on an infant with a positive SARS-CoV-2 RT-qPCR test, although the mother had negative SARS-CoV-2 RT-qPCR tests for milk, stool, and throat and vaginal swabs and repeated negative tests for serum IgG against SARS-CoV-2. Download Table S1, DOCX file, 0.1 MB. Copyright © 2021 Pace et al. 2021 Pace et al. This content is distributed under the terms of the Creative Commons Attribution 4.0 International license.

This paucity of rigorous methodology combined with inconsistency of viral RNA detection across studies has led to conflicting and changing recommendations regarding temporary separation of infants from mothers with COVID-19 and regarding whether infants should nurse directly at the breast or receive expressed milk from a bottle (10–13). Alongside the uncertainty about the risks of breastfeeding in the context of maternal COVID-19, it is well established that breastfeeding reduces the risk of a myriad of short- and long-term infectious and noninfectious conditions (14). Further, even a short delay in initiation of breastfeeding can interfere with the establishment of lactation (15) and increase risks of infant morbidity and mortality (16–18).

Many of the health-promoting effects of breastfeeding are due to the provision of passive immunity via immunoglobulins and other bioactive factors (e.g., lactoferrin), and previous studies have shown that milk-borne antibodies are produced in response to viral infection (19–22). However, few studies have examined the presence of antibodies to SARS-CoV-2 in human milk (23, 24). In one recent study, milk from 12 of 15 women who either had a laboratory-confirmed SARS-CoV-2 infection or were presumed infected with SARS-CoV-2 contained IgA that was reactive to the receptor binding domain (RBD) of the SARS-CoV-2 spike protein (24). Milk from all 15 women contained higher levels of IgA that was reactive to the full spike protein than milk collected prior to December 2019 (prepandemic). Serologic reactivity of antibodies in serum samples collected from healthy individuals and those infected with non-SARS common cold coronaviruses (ccCoVs) with SARS-CoV-2 have also been reported (25). This cross-reactivity is thought to stem from homology of the S2 domain of the spike (S) and nucleocapsid (N) proteins. The extent to which milk-borne antibodies have cross-reactivity to ccCoVs and whether these cross-reactive antibodies neutralize SARS-CoV-2 are currently not known (26).

The primary objective of this study was to determine whether SARS-CoV-2 can be detected in milk produced by, and on the breast skin of, women recently diagnosed with COVID-19 utilizing rigorous collection and analytical techniques. We also quantified anti-SARS-CoV-2 IgA and IgG in milk and the capacity of milk to neutralize SARS-CoV-2. Because subclinical mastitis has been associated with higher viral loads in milk (27), we also documented sodium-to-potassium ratios (Na/K) in milk, a biomarker of subclinical mastitis.

## RESULTS

### Participants and samples collected.

**FIG 1:**
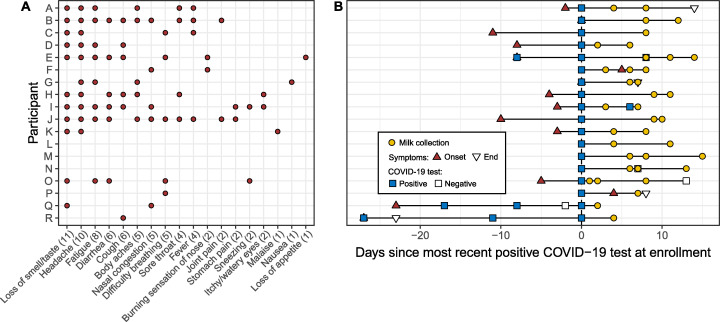
Overview of 18 participants’ COVID-19 signs and symptoms (A) and COVID-19 diagnostic testing and sampling (B). In panel A, counts for individual signs and symptoms are presented in parentheses. In panel B, the time “0” represents the most recent positive COVID-19 test at enrollment. Participants E, I, N, O, Q, and R had additional COVID-19 tests performed; all were positive except for N’s and O’s second test and Q’s third test (false-negative result), which were negative (open squares). Participants L, M, and N were asymptomatic during the study period, and participants F and P developed symptoms after first testing positive.

Eighteen women with a recent diagnosis of laboratory-confirmed COVID-19 participated in the study. On average, women were 34.2 ± 4.7 years old and 6.8 ± 7.8 months postpartum. Additional characteristics of study participants and their infants are presented in Table 1. Of the 18 participants, all but three had symptoms related to COVID-19, with the most common being loss of smell/taste (11/18), headache (10/18), and fatigue (7/18) (Fig. 1A). Two of the three participants who were asymptomatic throughout the course of the study were initially tested for routine surveillance prior to being admitted to the hospital for labor and delivery (participants L and M), while the other (participant N) was tested due to a potential occupational exposure. Within this cohort six infants were tested for COVID-19, two of whom had a positive result (infants of participants F and J). In both these cases, additional household members had confirmed positive COVID-19 tests. Among the four infants with negative COVID-19 tests (infants of participants E, I, L, and P), there were no additional household members with positive COVID-19 tests. Most infants (83%, 15/18) appeared well with no clinical signs of illness. Among the three infants with signs of COVID-19, one (participant A’s infant) had a case of diarrhea that resolved the same day; one (participant J’s infant and one of the two infants with a positive COVID-19 test) had a fever, cough, nasal congestion, low reactivity/appetite, and rash; and one (participant B’s infant) had a fever, cough, and sneezing and was irritable. Interestingly, the other infant with a positive COVID-19 test did not have any signs of illness. By the end of the study period, all infants’ signs of illness had subsided.

**TABLE 1:** Study participant characteristics (*n *= 18)^*a*^

Maternal characteristics (*n* = 18)		Infant characteristics (*n* = 18)	
Age (yr)	34.2 ± 4.7	Female	9 (50)
Race/ethnicity		Gestational age (wk)	38.6 ± 1.7
*Black, Hispanic*	1 (6)	Birth wt (g)	3,372 ± 560
*Black, non-Hispanic*	1 (6)	Birth length (cm)**	50.3 ± 2.7
*Pacific Islander*	1 (6)	Breastfeeding status	
*White, Hispanic*	1 (6)	*Exclusive*	5 (28)
*White, non-Hispanic*	14 (78)	*Mixed feeding*	13 (72)
Body mass index (kg/m^2^)*	28.9 ± 4.8	COVID-19 diagnostic test	6 (33)
*Normal/healthy*	5 (28)	*Negative result*	4 (67)
*Overweight*	7 (39)		
*Obese*	6 (33)		
Parity (no.)	1.9 ± 1.1		
Cesarean delivery	6 (33)		
Time postpartum (mo)	6.8 ± 7.8		
History of mastitis**	1 (7)		
Symptomatic at or prior to enrollment	14 (78)		
Developed symptoms after enrollment	1 (6)		
Asymptomatic	3 (16)		

a Categorical data are given as number of participants and, in parentheses, percentage of total. Continuous data are provided as means ± standard deviations. *, definitions put forth by the U.S. Centers for Disease Control and Prevention were used for body mass index categories. **, missing data from 1 individual.

We collected and analyzed 37 milk samples (Fig. 1B). Repeated samples were collected from 14 participants. Among women with clinical signs and/or symptoms of COVID-19 at enrollment or who developed them during the study, 6 provided samples before onset or within the first week of signs/symptoms, with the earliest sample collected 2 days prior to symptom(s) onset. This participant was initially tested for COVID-19 because of a close family exposure even though she was not currently symptomatic. Across all participants, the first sample was collected on average 12.0 ± 8.9 days after onset of signs and/or symptoms. Breast swabs were collected from 15 women, although participant F collected swabs prior to breast cleaning and then after milk collection (rather than before milk collection) (see Table S2 in the supplemental material).

10.1128/mBio.03192-20.4 TABLE S2 Breast skin swabs with evidence of SARS-CoV-2 RNA presence. A total of 35 sets of breast swabs (*n *= 70) were collected. Only samples that had any detectable signal are shown. *C_T_* values for RT-qPCR duplicates of each SARS-CoV-2 target are given. Samples with *C_T_* values acquired for both RT-qPCR duplicates of a given target are indicated in bold. nd = not detected. *For this subject, all swabs which were supposed to be collected after breast washing and prior to milk collections were collected after milk collections. Download Table S2, DOCX file, 0.04 MB. Copyright © 2021 Pace et al. 2021 Pace et al. This content is distributed under the terms of the Creative Commons Attribution 4.0 International license.

### SARS-CoV-2 RNA and Na/K in milk.

None of the milk contained detectable SARS-CoV-2 RNA. RT-qPCR findings were not modified by the milk fraction tested (i.e., whole milk or supernatant), and results were concordant between laboratories. Milk Na/K ratios ranged from 0.2 to 10.9 (0.5, median) with 12 (36%) samples having an elevated ratio (>0.6), suggesting subclinical mastitis in 9 participants.

### SARS-CoV-2 RNA on breast swabs.

Of the 70 swabs tested, eight had evidence of SARS-CoV-2 RNA (Table S2). One swab collected prior to breast washing tested conclusively positive with threshold cycle (*C_T_*) values of <40 in both duplicates for both the N1 and N2 targets. Two additional swabs collected prior to breast washing had detectable signal in both duplicates for one of the SARS-CoV-2 targets, but only one duplicate for the other target. Five swabs had detectable signal in just one duplicate for one target.

### Anti-SARS-CoV-2 IgA and IgG in milk.

**FIG 2:**
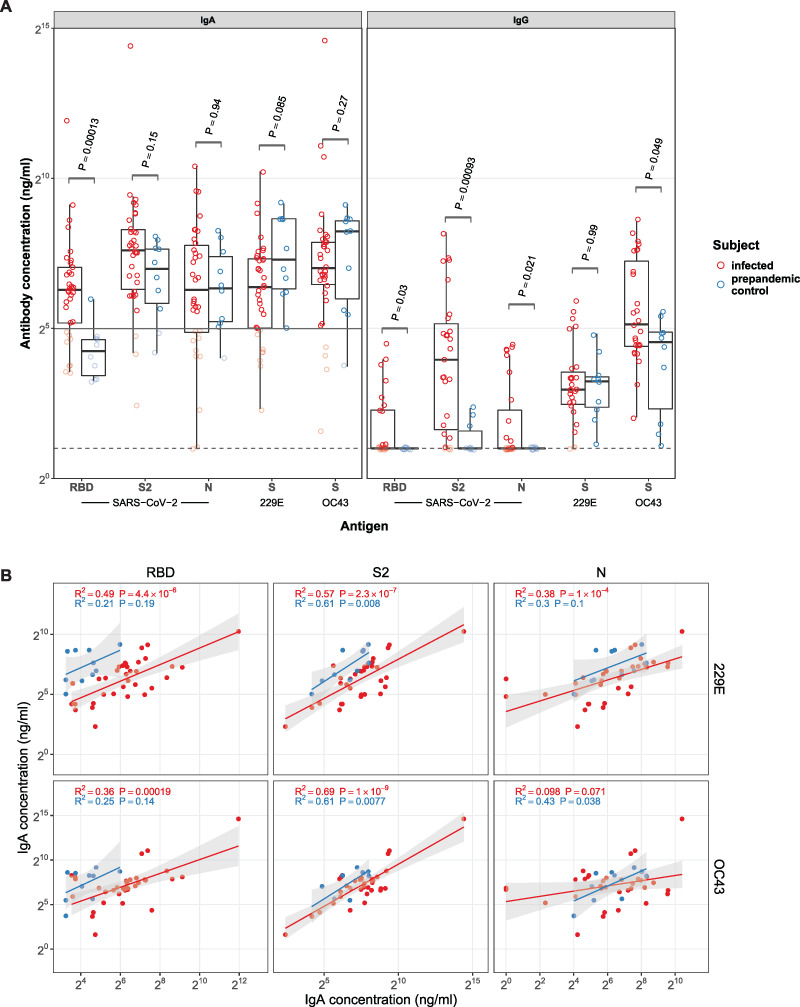
Milk antibody concentrations. Panel A shows IgA and IgG to coronavirus antigens in milk produced by COVID-19-infected (red) and healthy, prepandemic (blue) women. Antibody concentrations were measured using ELISA specific to the RBD and S2 domains of the spike and nucleocapsid (N) proteins of SARS-CoV-2 and S proteins from human coronaviruses 229E and OC43. The horizontal solid line in the IgA panel denotes the limit of antigen-specific binding that is defined as mean + 2× standard deviation of RBD-specific IgA signal in prepandemic controls. The dotted horizontal line denotes the ELISA limit of quantification. *P* values for the difference between infected and prepandemic controls are from the Wilcoxon signed-rank test. Milk from 44 mothers (34/10, infected/controls) was used for IgA testing, and milk from 37 mothers (27/10, infected/controls) was used for IgG testing. Panel B shows correlations between IgA concentrations to SARS-CoV-2 antigens RBD, S2, and N (*x* axis) and IgA concentrations to S proteins from 229E and OC43 (*y* axis) in milk produced by COVID-19-infected (red, *n *= 34) and healthy, prepandemic (blue, *n *= 10) women. A linear model was fitted to log-transformed IgA concentrations to give *r*-squared and *P* values as indicated. Model prediction (red or blue line as defined above) is shown with the 95% confidence interval (gray shading).

Of the milk samples tested, we found that 76% (26/34) contained SARS-CoV-2-specific IgA and 80% (22/27) contained SARS-CoV-2-specific IgG. Concentrations of anti-SARS-CoV-2 IgA were consistently higher than those of IgG (Fig. 2A). Milk produced by women with COVID-19 had higher anti-RBD IgA and IgG concentrations than milk collected from women before the pandemic (*P* = 0.00013 and *P* = 0.03, respectively). This pattern was also evident for anti-spike S2 subunit (S2) and antinucleocapsid (N) IgG (*P* = 0.00093 and *P* = 0.021, respectively), but not IgA. There were no significant differences in IgA and IgG to the full-length S proteins of ccCoV 229E and IgA to those of OC43 between milk produced by women with COVID-19 and milk produced before the pandemic. Milk produced by infected mothers, however, contained higher levels of IgG to OC43 (*P* = 0.049) than did prepandemic milk. Concentrations of IgA to SARS-CoV-2 antigens correlated well with ccCoV antigens, suggesting significant cross-reactivity of antibodies to ccCoV spike protein and SARS-CoV-2 S2 fragment (Fig. 2B). The correlation was particularly high in milk produced by women with COVID-19 and between spike proteins, suggesting that IgA to SARS-CoV-2 S2 was also reactive to ccCoVs’ spike proteins and may be a recall response from prior exposure to ccCoVs.

### Neutralizing capacity of milk.

**FIG 3:**
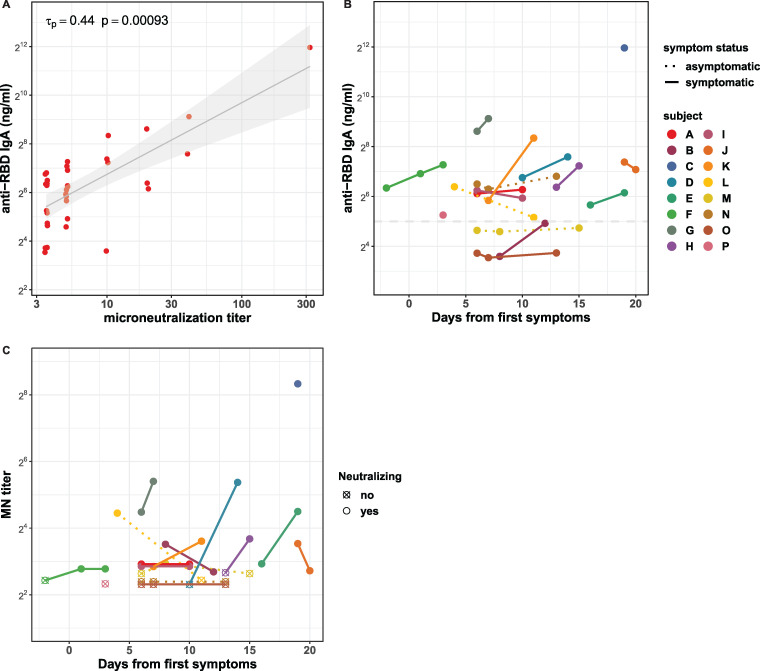
Correlation of milk IgA concentrations with microneutralization titers in milk produced by women with COVID-19 (*n *= 34). Panel A shows the correlation between concentrations of IgA specific to SARS-CoV-2 RBD and microneutralization (MN) titers. Kendall rank correlation τ_p_ and associated *P* value are as shown. A linear model was fitted to log-transformed IgA concentrations and MN titers and is shown (black line) with 95% confidence interval (gray shading) for visualization purposes only. Time courses of anti-RBD IgA concentrations and MN titers are shown in panels B and C, respectively. Each color and corresponding point or set of connected points represent one participant. Time of collection is indicated on the *x* axis as days from the appearance of first symptoms or, for asymptomatic cases, from the time of diagnosis. Antibody concentration is indicated on the *y* axis of panel B, and MN titer is indicated on the *y* axis of panel C. Asymptomatic participants are indicated using a dotted line connecting points. The horizontal dashed line in panel B denotes the limit of antigen-specific binding as defined in Fig. 2A.

A total of 21 of 34 milk samples (62%) collected from women with COVID-19 were found to neutralize SARS-CoV-2 infectivity *in vitro*, whereas none of the prepandemic samples were able to do so. Although microneutralization (MN) titers correlated with concentrations of IgA to all SARS-CoV-2 antigens tested (Fig. 3A and Fig. S1), in a multivariable regression model that included all antigen targets as independent variables, only the SARS-CoV-2 RBD had a significant β (*P* = 0.0125), consistent with neutralization primarily by anti-RBD antibodies. Consequently, an analysis of sequential milk samples collected from study participants identified increases in anti-RBD IgA concentrations in 9 of 15 cases, and 5 of those were associated with elevated microneutralization titers (Fig. 3B). Anti-RBD IgA was also correlated with anti-RBD IgG, resulting in an apparent correlation of anti-RBD IgG and MN titer (data not shown). However, anti-RBD IgG titers as low as those found here, however, were not neutralizing in our previous study (28). It is noteworthy that at least one milk sample provided by subjects B and M exhibited neutralizing capacity even though anti-RBD IgA levels were not above those of prepandemic samples. These same samples also did not have detectable anti-RBD IgG.

10.1128/mBio.03192-20.2 FIG S1 Correlation of SARS-CoV-2-specific IgA antibody concentrations with neutralization titers (*n *= 34). Kendall rank correlation τ_p_ and associated *P* value are as shown. A linear model was fitted to log-transformed IgA concentrations and MN titers and is shown (black line) with 95% confidence interval (gray shading) for visualization purposes only. Download FIG S1, EPS file, 3.0 MB. Copyright © 2021 Pace et al. 2021 Pace et al. This content is distributed under the terms of the Creative Commons Attribution 4.0 International license.

## DISCUSSION

Although human milk is considered the best source of nutrition for most infants, the onset of the global COVID-19 pandemic and our lack of understanding of SARS-CoV-2 transmission have caused confusion around whether infected mothers should be temporarily separated from their infants, as well as whether breastfeeding should be initiated and/or continued during maternal COVID-19 illness. In this prospective study, we collected milk and breast swabs from women with COVID-19 and tested them for the presence of SARS-CoV-2 RNA. We also analyzed the milk for IgA and IgG targeting SARS-CoV-2 and the ability of the samples to neutralize SARS-CoV-2 and thereby reduce infectivity. Milk samples collected prior to the pandemic were analyzed as reference controls.

Using methods validated and replicated across two laboratories, and consistent with most previous reports (5–9) (see Table S1 in the supplemental material), we did not detect SARS-CoV-2 RNA in any of the milk samples. Although a single swab collected from the breast before it was cleaned was found conclusively to contain SARS-CoV-2 RNA, a swab collected after the breast was washed did not. Although study participants wore masks and gloves, the presence of SARS-CoV-2 RNA on this breast swab, and the indeterminate detection on 7 other swabs, may help explain the findings of other studies that have reported detecting viral RNA in some milk samples (i.e., contamination of milk via skin and/or respiratory droplets) (4, 29, 30). It is reassuring that a majority of the infants (83%, 15/18) appeared healthy with no signs of illness, including two whose mothers had detectable viral RNA on their breast skin. Nonetheless, the infants of three participants with viral RNA detected on their breast skin were observed to have at least one symptom associated with COVID-19, although only one of the three infants had a diagnostic test performed (which came back positive). However, it should be noted that in these three cases additional household members were confirmed to have COVID-19, making it difficult to attribute their symptoms or mode of transmission, in the case of the positive infant, to breastfeeding.

It is also noteworthy that several of the women in our study had evidence of subclinical mastitis, which has previously been shown to be positively related to viral load in milk produced by HIV-infected women (27). Nonetheless, we detected no SARS-CoV-2 RNA in the milk produced by any participant in our study. This suggests that there is likely no relationship between subclinical mastitis and the presence of SARS-CoV-2 RNA in a woman’s milk. Together, our results suggest that milk does not appear to act as a vehicle for mother-to-child transmission of SARS-CoV-2 in women with mild-to-moderate COVID-19, although viral exposure via breast skin is possible. Our lack of detection of viral RNA on the breast after washing supports existing recommendations for women to take precautions during breastfeeding and/or expression of milk (e.g., practicing respiratory and hand hygiene, cleaning pump parts prior to and after use) to reduce the potential for viral transmission.

Importantly, human milk contains a wide variety of immunoglobulins, of which IgA represents the majority (80 to 90%) (31, 32). In our study, we detected anti-SARS-CoV-2 antibodies in milk, primarily IgA but also IgG, albeit at lower concentrations than those reported for serum of COVID-19 patients during convalescent phase (28). That same study also noted specific cross-reactivity between the S2 subunits of SARS-CoV-2 and the ccCoV OC43. We also observed that concentrations of anti-SARS-CoV-2 S2 antibodies correlated strongly with those of the other tested ccCoV spike proteins in milk produced by study participants and those produced prior to the pandemic. This pattern of cross-reactivity may reflect structural similarity among the proteins and likely reflects a recall response from prior exposure to ccCoV. However, generation of RBD-reactive antibodies likely requires activation of antigen-specific naive B-cell populations, because the RBD of the SARS-CoV-2 spike protein shares little sequence homology with other ccCoVs.

It is noteworthy that while IgA to SARS-CoV-2 S2 subunit and N protein and ccCoVs is present in prepandemic samples, IgG levels to the same antigens except the S protein of 229E are increased in infected mothers. This pattern of increase in milk IgG reactivity is identical to the one we described for serum previously (28). The difference between IgA and IgG could be due to the shorter lifetime of systemic IgG-secreting B cells versus long-lived submucosal IgA-secreting B cells in the mammary gland following a previous exposure to ccCoVs. This is also consistent with similar apparent difference in specificity of milk IgA versus IgG for influenza virus (33).

Of particular note, we are the first to report that milk produced by women with COVID-19 is able to neutralize SARS-CoV-2 infectivity. While neutralization titer correlated with concentration of RBD-reactive IgA, neutralization was detected in three samples with low RBD-reactive IgA and without detectable RBD-reactive IgG. This may suggest other factors present in milk produced by infected mothers or higher-affinity milk IgA enhancing SARS-CoV-2 neutralization.

While the detection of SARS-CoV-2 RNA in milk and/or on the breast is of concern, it does not necessarily indicate the presence of viable or infectious virus. In the only study that has assessed the viability of SARS-CoV-2 in milk, a single milk sample positive for SARS-CoV-2 RNA did not contain replication-competent virus (30). Unfortunately, in our study we were unable to determine the viability of SARS-CoV-2 in any of the breast swabs positive for SARS-CoV-2 RNA because the entire sample was needed for RNA detection. Future studies should determine the viability of any SARS-CoV-2 found in milk and/or on the breast.

Our study has several important strengths, including the use of rigorous collection methods, close temporal proximity of sample collections to COVID-19 diagnosis, validation of analytical methods for human milk, replication of RT-qPCR analyses across laboratories, and analysis of both risks and benefits of milk constituents. We also acknowledge that this study has limitations. For instance, as samples were self-collected and stored in participant freezers, it remains possible, although we believe unlikely, that sample quality and/or contamination may have influenced the results. However, our preliminary testing of various storage and temperature conditions would indicate that the SARS-CoV-2 RNA is robust to similar conditions. Most samples were also collected from women after onset of symptoms, limiting generalizability to presymptomatic women. Differences in symptom onset and time of infection may also be related to the variation in antibody concentrations that we observed. Additionally, no participant was hospitalized due to COVID-19. As disease severity may be related to viral titer (34), it is possible that milk produced by individuals with more severe COVID-19 could contain SARS-CoV-2. The short duration of the follow-up period also does not allow characterization of the durability of the milk IgA and IgG responses. Initial reports on serum IgG response may suggest a relatively short-lived response (35, 36), and no data exist on the presence of long-lived memory B cells in the context of SARS-CoV-2. Milk IgA, representing a mucosal response, may have its own pattern of durability.

### Conclusions.

We did not detect SARS-CoV-2 RNA in milk produced by women with mild-to-moderate COVID-19. Moreover, we demonstrated that milk contains anti-SARS-CoV-2 antibodies and that their concentrations are correlated with milk’s ability to effectively neutralize SARS-CoV-2 infectivity. We found evidence of SARS-CoV-2 on the areola/nipple region of several women, but it is unclear whether this RNA reflects viable virus. As such, our data do not suggest that infected women should systematically wash their breasts prior to breastfeeding or expressing milk. However, and in support of recommendations put forth by the WHO, if a mother who is confirmed/suspected to have COVID-19 has just coughed over her exposed breast, she should gently wash the breast with soap and warm water before feeding (37). Taken together with the well-documented benefits of breastfeeding to maternal and infant health, our data support recommendations to encourage breastfeeding in women with mild-to-moderate COVID-19 illness.

## MATERIALS AND METHODS

### Experimental design and clinical data collection.

This prospective study was carried out using a repeated-measures, longitudinal design. To be eligible, women needed to be ≥18 years of age, to be lactating, and to have received a positive test result for COVID-19 in the previous 8 days. Subjects were recruited through social media, word of mouth, and the assistance of national maternal and child health organizations and collaborating hospitals. All participants gave informed consent, and procedures were approved by the Institutional Review Boards at the University of Idaho (20-056, 20-060), the University of Rochester Medical Center (1507), and Brigham and Women’s Hospital (2020P000804). Surveys were administered by telephone to ascertain timing of maternal/infant COVID-19 symptoms, reproductive history, breast health, breastfeeding practices, demographics, and anthropometrics.

### Milk and breast swabs.

All collection kits were assembled aseptically by study personnel wearing masks and gloves and were individually packaged to reduce potential contamination. Mothers were instructed in clean techniques to obtain samples, including use of gloves and masks. Milk and swabs of the nipple/areola (“breast swabs”) were self-collected either in participants’ homes (with virtual assistance provided by study personnel) or at a hospital (participants Q and R were admitted to the postpartum unit at the time of sample collection). Breast swabs were collected before and after washing the breast with soap and water and prior to milk collection. Women collected up to 30 ml of milk using the provided sterile manual breast pump (Harmony; Medela) and sterile collection containers. Details regarding sample collection are provided in Text S1 in the supplemental material.

10.1128/mBio.03192-20.1 TEXT S1 Milk and swab collection methods, RNA extraction and RT-qPCR assay details, validation of RT-qPCR assay, and sample processing for microneutralization assays. Download Text S1, DOCX file, 0.04 MB. Copyright © 2021 Pace et al. 2021 Pace et al. This content is distributed under the terms of the Creative Commons Attribution 4.0 International license.

Following collection, samples were immediately frozen in the subject’s freezer until being shipped in a cooler containing frozen cold packs to the University of Idaho (UI) or University of Rochester Medical Center (URMC). Samples collected from subjects Q and R were frozen at −80°C and shipped on dry ice to UI. Once received, samples were processed immediately for RNA extraction and/or stored at −80°C until further analysis. As needed, samples were shipped on dry ice between UI and URMC. Milk samples collected prior to December 2019 from 10 healthy women located in the Rochester, NY, area for general assay development purposes were used as prepandemic control samples.

### SARS-CoV-2 RNA.

Details related to RNA extraction and assay validation are provided in the Text S1. Briefly, RNA was extracted from milk (at both UI and URMC), breast swabs (UI), and extraction controls (at both UI and URMC) using the Quick-DNA/RNA Viral MagBead kit (Zymo Research, Irvine, CA) with addition of the DNase I treatment on extracted nucleic acids following the manufacturer’s protocol. Detection of SARS-CoV-2 viral RNA in milk was independently determined in both UI and URMC laboratories using the CDC-designed 2019-nCoV RT-qPCR assay (38), validated in both laboratories for use with human milk. Per the CDC protocol, samples with *C_T_* values of <40 were considered positive.

### Antibodies.

Concentrations of milk-borne IgA and IgG reactive to the SARS-CoV-2 spike (both S2 subunit and RBD) and nucleocapsid (N) proteins and full-length spike proteins of common cold coronaviruses 229E and OC43 were measured in milk samples by ELISA as previously described (39). Briefly, Nunc MaxiSorp 96-well plates (Thermo Fisher, Waltham, MA) were coated with optimized concentrations of antigens (1 to 5 μg/ml) overnight at 4°C. Coated plates were blocked for 1 h before the addition of serial 2-fold dilutions of samples. After 2 h of incubation at room temperature, plates were washed and bound IgG and IgA were detected with alkaline phosphatase-conjugated anti-human IgG (clones MT78 and MT57, respectively; Mabtech, Stockholm, Sweden) and anti-human IgA. Bound antigen-specific antibodies were detected by adding *p*-nitrophenyl phosphate substrate (Thermo Fisher). Absorbance was read at 405 nm after color development. A weight-based concentration method was used to assign antigen-specific antibody titers in test samples (39, 40).

### SARS-CoV-2 neutralization.

The neutralizing activity of milk against SARS-CoV-2 was measured by microneutralization (MN) assay. Briefly, duplicates of delipidated milk were serially diluted 2-fold in virus diluent and incubated with 100 50% tissue culture infective doses (TCID_50_) of SARS-CoV-2 virus (Hong Kong/VM20001061/2020 isolate) in 96-well flat-bottomed plates for 1 h at 37°C. After incubation, Vero E6/TMPRSS2 cells (kindly provided by Yoshihiro Kawaoka, National Institute of Infectious Diseases, Japan; 25,000 cells/well) (41) were added to the virus-sample mixtures. Plates were incubated for 48 h at 37°C, when a cytopathic effect was evident in virus-only control wells. Cells were then fixed with 6% paraformaldehyde for 30 min and washed and stained with crystal violet for 1 h. The MN titer was identified as the highest dilution of sample that showed 50% neutralization based on the appearance of the stained cell monolayer compared with the virus control well.

### Sodium and potassium.

For milk samples with sufficient volume (33 of 37), sodium (Na) and potassium (K) concentrations were quantified in 200 μl of milk using LAQUAtwin ion selective meters (Na-11 and K-11, respectively; Horiba Ltd., Kyoto, Japan). Prior to use, each meter was conditioned and calibrated according to vendor specifications. After each measurement, meters were rinsed with Nanopure water, wiped dry, and allowed to reach zero. A Na-to-K ratio (Na/K) of >0.6 was interpreted to indicate subclinical mastitis (42, 43).

### Statistical analysis.

Except where noted, all statistical analyses were performed using R (version 3.6.1). Statistical testing of antibody concentrations and MN titers was performed using nonparametric tests. Linear regression was performed using either the lm() or rlm() function in R as appropriate. Significance was declared at *P* < 0.05.

### Data availability.

The data sets generated during and/or analyzed during the current study are available from the corresponding author on reasonable request.
