# Supplementary material for: Characterization of SARS-CoV-2 RNA, Antibodies, and Neutralizing Capacity in Milk Produced by Women with COVID-19
Source: mBio. 2021 Feb 9;12(1):e03192-20. doi: 10.1128/mBio.03192-20 (PMC7885115; doi:10.1128/mBio.03192-20)
Supplement: TABLE S1 [file mBio.03192-20-st001.docx]

| **Study** | **Location** | **Participants, n** | **Participants with**  **SARS-CoV-2 RNA positive milk, n (%)** |
| --- | --- | --- | --- |
| **AlZaghal et al., 2020**^1^ | Jordan | 1 | 0 (0) |
| **Bastug et al., 2020**^2^ | Turkey | 1 | 1 (100) |
| **Bertino et al., 2020**^3^ | Italy | 14 | 1 (7) |
| **Buonsenso et al., 2020**^4^***** | Italy | 2 | 1 (50) |
| **Chambers et al., 2020**^5^ | USA | 18 | 1 (6) |
| **Chen et al., 2020**^6^ | China | 6 | 0 (0) |
| **Carosso et al., 2020**^7^ | Italy | 1 | 0 (0) |
| **Costa et al., 2020**^8^***** | Italy | 2 | 1 (50) |
| **Cui et al., 2020**^9^ | China | 1 | 0 (0) |
| **De Socio et al., 2020**^10^ | Italy | 1 | 0 (0) |
| **Deng et al., 2020**^11^ | China | 6 | 0 (0) |
| **Dong, Chi et al., 2020**^12^ | China | 1 | 0 (0) |
| **Dong, Tian et al., 2020**^13^ | China | 1 | 0 (0) |
| **Fan et al., 2020**^14^ | China | 2 | 0 (0) |
| **Fenizia et al., 2020**^15^ | Italy | 11 | 1 (9) |
| **Gao et al., 2020**^16^ | China | 12 | 0 (0) |
| **Gidlof et al., 2020**^17^ | Sweden | 1 | 0 (0) |
| **Groß et al., 2020**^18^ | Germany | 2 | 1 (50) |
| **Han et al., 2020**^19^ | Korea | 1 | 0 (0) |
| **Hinojosa-Velasco et al., 2020**^20^ | Mexico | 1 | 1 (100) |
| **Kalafat et al., 2020**^21^ | Turkey | 1 | 0 (0) |
| **Kam et al., 2020**^22^ | Singapore | 1 | 0 (0) |
| **Kirtsman et al., 2020**^23^ | Canada | 1 | 1 (100) |
| **Lang et al., 2020**^24^ | China | 1 | 0 (0) |
| **Lei et al., 2020**^25^ | China | 4 | 0 (0) |
| **Li, Hu, et al., 2020**^26^ | China | 1 | 0 (0) |
| **Li, Zhao, et al., 2020**^27^ | China | 1 | 0 (0) |
| **Liu, Wang, Li, et al., 2020**^28^ | China | 10 | 0 (0) |
| **Liu, Wang, Zhang, et al., 2020**^29^ | China | 2 | 0 (0) |
| **Lugli et al. 2020**^30^ | Italy | 1 | 1 (100) |
| **Luo et al., 2020**^31^ | China | 23 | 0 (0) |
| **Mao et al., 2020**^32^ | China | 1 | 0 (0) |
| **Marin Gabriel et al., 2020**^33^ | Spain | 7 | 0 (0) |
| **Menter et al., 2020**^34^ | Switzerland | 4 | 0 (0) |
| **Pace et al., 2020 (present study)** | USA | 18 | 0 (0) |
| **Peng, Wang et al., 2020**^35^ | China | 1 | 0 (0) |
| **Peng, Zhu et al., 2020**^36^ | China | 16 | 0 (0) |
| **Perrone et al., 2020**^37^ | Italy | 1 | 0 (0) |
| **Piersigilli et al., 2020**^38^ | Belgium | 1 | 0 (0) |
| **Sahin et al., 2020**^39^ | Turkey | 29 | 0 (0) |
| **Salvatori et al., 2020**^40^ | Italy | 2 | 0 (0) |
| **Schoenmakers et al., 2020**^41^ | Netherlands | 1 | 0 (0) |
| **Slaats et al., 2020**^42^****** | Netherlands | 1 | 0 (0) |
| **Tam et al., 2020**^43^ | Australia | 1 | 1 (100) |
| **Walczak et al., 2020**^44^ | Australia | 1 | 0 (0) |
| **Wang et al., 2020**^45^ | China | 1 | 0 (0) |
| **Wu et al., 2020**^46^ | China | 3 | 1 (33) |
| **Xiong et al., 2020**^47^ | China | 1 | 0 (0) |
| **Yan et al., 2020**^48^ | China | 12 | 0 (0) |
| **Yu et al., 2020**^49^ | China | 1 | 0 (0) |
| **Yuehua et al., 2020**^50^ | China | 1 | 0 (0) |
| **Zhao et al., 2020**^51^ | China | 4 | 0 (0) |
| **Zhu et al., 2020**^52^ | China | 5 | 1 (20) |
| **TOTAL** | - | 241 | 12 (5) |

**Supplemental Table 1.** Studies examining human milk for evidence of SARS-CoV-2 RNA via RT-qPCR. *Buonsenso et al., 2020 and Costa et al., 2020 reported on the same participants and are both included for completeness, but not duplicated in the total counts. **Slaats et al., 2020 reported on an infant with a positive SARS-CoV-2 RT-qPCR test, although the mother had negative SARS-CoV-2 RT-qPCR tests for milk, stool, throat and vaginal swabs, and repeated negative tests for serum IgG against SARS-CoV-2.

**References**

1. AlZaghal LA, AlZaghal N, Alomari SO, Obeidat N, Obeidat B, Hayajneh WA. Multidisciplinary team management and cesarean delivery for a Jordanian woman infected with SARS-COV-2: A case report. Case Rep Women's Health 2020;27:e00212.

2. Bastug A, Hanifehnezhad A, Tayman C, et al. Virolactia in an asymptomatic mother with COVID-19. Breastfeed Med 2020;15(8):488–91.

3. Bertino E, Moro GE, De Renzi G, et al. Detection of SARS-CoV-2 in milk From COVID-19 positive mothers and follow-up of their infants. Front Pediatr 2020;8:809.

4. Buonsenso D, Costa S, Sanguinetti M, et al. Neonatal late onset infection with severe acute respiratory syndrome coronavirus 2. Am J Perinatol 2020;37(08):869–72.

5. Chambers C, Krogstad P, Bertrand K, et al. Evaluation for SARS-CoV-2 in breast milk from 18 infected women. JAMA 2020;324(13):1347–8.

6. Chen H, Guo J, Wang C, et al. Clinical characteristics and intrauterine vertical transmission potential of COVID-19 infection in nine pregnant women: a retrospective review of medical records. Lancet 2020;395(10226):809–15.

7. Carosso A, Cosma S, Borella F, et al. Pre-labor anorectal swab for SARS-CoV-2 in COVID-19 pregnant patients: is it time to think about it? Eur J Obstet Gynecol Reprod Biol 2020;249:98–9.

8. Costa S, Posteraro B, Marchetti S, et al. Excretion of SARS-CoV-2 in human breast milk. Clin Microbiol Infect 2020;26(10):1430–2.

9. Cui Y, Tian M, Huang D, et al. A 55-day-old female infant infected with 2019 novel coronavirus disease: presenting with pneumonia, liver injury, and heart damage. J Infect Dis 2020;376:584–7.

10. De Socio GV, Malincarne L, Arena S, et al. Delivery in asymptomatic Italian woman with SARS-CoV-2 infection. Mediterr J Hematol Infect Dis 2020;12(1):e2020033.

11. Deng G, Zeng F, Zhang L, Chen H, Chen X, Yin M. Characteristics of pregnant COVID-19 patients with liver injury. J Hepatol 2020;S0168-8278(20)30395-0.

12. Dong Y, Chi X, Hai H, et al. Antibodies in the breast milk of a maternal woman with COVID-19. Emerg Microbes Infect 2020;9(1):1467–9.

13. Dong L, Tian J, He S, et al. Possible vertical transmission of SARS-CoV-2 from an infected mother to her newborn. JAMA 2020;323(18):1846–8.

14. Fan C, Lei D, Fang C, et al. Perinatal transmission of COVID-19 associated SARS-CoV-2: should we worry? Clin Infect Dis 2020;:ciaa226.

15. Fenizia C, Biasin M, Cetin I, et al. Analysis of SARS-CoV-2 vertical transmission during pregnancy. Nat Commun 2020;11(1):5128.

16. Gao X, Wang S, Zeng W, et al. Clinical and immunologic features among COVID-19-affected mother-infant pairs: antibodies to SARS-CoV-2 detected in breast milk. New Microbes and New Infections 2020;37:100752.

17. Gidlöf S, Savchenko J, Brune T, Josefsson H. COVID‐19 in pregnancy with comorbidities: More liberal testing strategy is needed. Acta Obstet Gynecol Scand 2020;99(7):948–9.

18. Groß R, Conzelmann C, Müller JA, et al. Detection of SARS-CoV-2 in human breastmilk. The Lancet 2020;395(10239):1757–8.

19. Han MS, Seong M-W, Heo EY, et al. Sequential analysis of viral load in a neonate and her mother infected with SARS-CoV-2. Clin Infect Dis 2020;35:e124.

20. Hinojosa-Velasco A, de Oca PVB-M, García-Sosa LE, et al. A case report of newborn infant with severe COVID-19 in Mexico: Detection of SARS-CoV-2 in human breast milk and stool. Int J Infect Dis 2020;100:21–4.

21. Kalafat E, Yaprak E, Cinar G, et al. Lung ultrasound and computed tomographic findings in pregnant woman with COVID‐19. Ultrasound Obstet Gynecol 2020;55(6):835–7.

22. Kam K-Q, Yung CF, Cui L, et al. A well infant with coronavirus disease 2019 (COVID-19) with High Viral Load. Clin Infect Dis 2020;361:1701.

23. Kirtsman M, Diambomba Y, Poutanen SM, et al. Probable congenital SARS-CoV-2 infection in a neonate born to a woman with active SARS-CoV-2 infection. CMAJ 2020;192(24):E647–50.

24. Lang G-J, Zhao H. Can SARS-CoV-2-infected women breastfeed after viral clearance? J Zhejiang Univ Sci B 2020;21(5):405–7.

25. Lei D, Wang C, Li C, et al. Clinical characteristics of COVID-19 in pregnancy: analysis of nine cases. Chinese Journal of Perinatal Medicine

26. Li Y, Hu Y, Yu Y, et al. Positive result of Sars‐Cov‐2 in faeces and sputum from discharged patient with COVID‐19 in Yiwu, China. J Med Virol 2020;:jmv.25905.

27. Li Y, Zhao R, Zheng S, et al. Lack of vertical transmission of severe acute respiratory syndrome coronavirus 2, China. Emerging Infect Dis 2020;26(6):727.

28. Liu W, Wang J, Li W, Zhou Z, Liu S, Rong Z. Clinical characteristics of 19 neonates born to mothers with COVID-19. Front Med 2020;14(2):193–8.

29. Liu W, Wang Q, Zhang Q, Chen L, Chen J, Zhang B. Coronavirus disease 2019 (COVID-19) during pregnancy: A case series. Preprints 2020;

30. Lugli L, Bedetti L, Lucaccioni L, et al. An aninfected preterm newborn inadvertently fed SARS-CoV-2–positive breast milk. Pediatrics 2020;:e2020004960.

31. Luo Q, Chen L, Yao D, et al. Safety of breastfeeding in mothers with SARS-CoV-2 infection. medRxiv 2020;:1–24.

32. Mao L-J, Xu J, Xu Z-H, et al. A child with household transmitted COVID-19. BMC Infect Dis 2020;20(1):727.

33. Marín Gabriel MÁ, Malalana Martínez AM, Marín Martínez ME, Anel Pedroche J. Negative transmission of SARS-CoV-2 to hand-expressed colostrum from SARS-CoV-2-positive mothers. Breastfeed Med 2020;15(8):492–4.

34. Menter T, Mertz KD, Jiang S, et al. Placental Pathology Findings during and after SARS-CoV-2 Infection: Features of Villitis and Malperfusion. Pathobiology 2020;:1–9.

35. Peng Z, Wang J, Mo Y, et al. Unlikely SARS-CoV-2 vertical transmission from mother to child: A case report. J Infect Public Health 2020;13(5):818–20.

36. Peng S, Zhu H, Yang L, et al. A study of breastfeeding practices, SARS-CoV-2 and its antibodies in the breast milk of mothers confirmed with COVID-19. SSRN Journal 2020;

37. Perrone S, Giordano M, Meoli A, et al. Lack of viral transmission to preterm newborn from a COVID‐19 positive breastfeeding mother at 11 days postpartum. J Med Virol 2020;0002(20):319149.

38. Piersigilli F, Carkeek K, Hocq C, et al. COVID-19 in a 26-week preterm neonate. Lancet Child Adolesc Health 2020;4(6):476–8.

39. Sahin D, Tanacan A, Erol SA, et al. A pandemic center’s experience of managing pregnant women with COVID‐19 infection in Turkey: A prospective cohort study. Int J Gynaecol Obstet 2020;:ijgo.13318.

40. Salvatori G, De Rose DU, Concato C, et al. Managing COVID-19-positive maternal-infant dyads: an italian experience. Breastfeed Med 2020;15(5):347–8.

41. Schoenmakers S, Snijder P, Verdijk R, et al. SARS-CoV-2 placental infection and inflammation leading to fetal distress and neonatal multi-organ failure in an asymptomatic woman. medRxiv 2020;:1–18.

42. Slaats MALJ, Versteylen M, Gast KB, et al. Case report of a neonate with high viral SARSCoV-2 loads and long-term virus shedding. J Infect Public Health 2020;

43. Tam PCK, Ly KM, Kernich ML, et al. Detectable severe acute respiratory syndrome coronavirus 2 (SARS-CoV-2) in human breast milk of a mildly symptomatic patient with coronavirus disease 2019 (COVID-19). Clin Infect Dis 2020;:ciaa673.

44. Walczak A, Wilks K, Shakhovskoy R, et al. COVID-19 in a complex obstetric patient with cystic fibrosis. Infect Dis Health 2020;25(4):239–41.

45. Wang S, Guo L, Chen L, et al. A case report of neonatal COVID-19 infection in China. Clin Infect Dis 2020;348:1953.

46. Wu Y, Liu C, Dong L, Chen Y, Liu J, Zhang C. Viral shedding of COVID-19 in pregnant women. SSRN Journal 2020;

47. Xiong X, Wei H, Zhang Z, et al. Vaginal delivery report of a healthy neonate born to a convalescent mother with COVID­‐19. J Med Virol 2020;:e204621–3.

48. Yan J, Guo J, Fan C, et al. Coronavirus disease 2019 in pregnant women: a report based on 116 cases. Am J Obstet Gynecol 2020;223(1):111.e1–111.e14.

49. Yu Y, Li Y, Hu Y, Li B, Xu J. Breastfed 13 month-old infant of a mother with COVID-19 pneumonia: a case report. International Breastfeeding Journal 2020;15(1):68.

50. Yuehua Z, Daojiong L, Meifang X, et al. A case of three-month-old infant with new coronavirus infection. Chinese Journal of Pediatrics 2020;58(3):182–4.

51. Zhao Y, Shang Y, Ren Y, et al. Omics study reveals abnormal alterations of breastmilk proteins and metabolites in puerperant women with COVID-19. Sig Transduct Target Ther 2020;5(1):473.

52. Zhu C, Liu W, Su H, et al. Breastfeeding risk from detectable severe acute respiratory syndrome coronavirus 2 in breastmilk. J Infect 2020;81(3):452–82.
