# Supplementary material for: Characterization of SARS-CoV-2 RNA, Antibodies, and Neutralizing Capacity in Milk Produced by Women with COVID-19
Source: mBio. 2021 Feb 9;12(1):e03192-20. doi: 10.1128/mBio.03192-20 (PMC7885115; doi:10.1128/mBio.03192-20)
Supplement: TABLE S2 [file mBio.03192-20-st002.docx]

|  |  | Ct values for SARS-CoV-2 RT-qPCR targets | |
| --- | --- | --- | --- |
| Participant | Collection | Before Breast Washing | After Breast Washing |
| A | First  Second | **N1:** **34.9/35.2**, N2: 37.0/nd  N1: nd, N2: 37.2/nd | nd  nd |
| B | First  Second | **N1: 30.4/30.7, N2: 30.4/30.9**  **N1:** **34.0/34.5**, N2: 36.1/nd | nd  nd |
| F* | First  Second  Third | nd  N1: nd; N2: 38.0/nd  nd | N1: 36.1/nd; N2: nd  nd  nd |
| G | First  Second | N1: 35.9/nd; N2: nd  nd | nd  nd |
| J | First  Second | N1: 36.0/nd; N2: nd  nd | nd  nd |

**Supplemental Table 2.** **Breast skin swabs with evidence of SARS-CoV-2 RNA presence**. A total of 35 sets of breast swabs (*n* = 70) were collected. Only samples that had any detectable SARS-CoV-2 signal are shown. Ct values for RT-qPCR duplicates of each SARS-CoV-2 target are given. Samples with Ct values acquired for both RT-qPCR duplicates of a given target are indicated in bold. nd = not detected. *For this subject, all swabs which were supposed to be collected after breast washing and prior to milk collections were collected after milk collections.
