# Supplementary material for: Characterization of SARS-CoV-2 RNA, Antibodies, and Neutralizing Capacity in Milk Produced by Women with COVID-19
Source: mBio. 2021 Feb 9;12(1):e03192-20. doi: 10.1128/mBio.03192-20 (PMC7885115; doi:10.1128/mBio.03192-20)
Supplement: TEXT S1 [file mBio.03192-20-s0001.docx]

**Supplemental text: milk and swab collection methods, RNA extraction and RT-qPCR assay details, validation of RT-qPCR, and sample processing for microneutralization assays**

1. Milk and breast swab samples collected at home
2. Milk samples collected in hospitals
3. RNA extraction
4. SARS-CoV-2 RT-qPCR assay
5. Validation of SARS-CoV-2 RT-qPCR for use with human milk
6. Sample processing for ELISA and microneutralization (MN) assays

**1. Milk and breast swab samples collected at home**

Prior to each milk collection, women were asked to put on a face mask and gloves and open and assemble the provided sterile manual breast pump (Harmony, Medela). After assembling the pump, women removed their gloves, washed their hands with soap and warm water for at least 20 sec, and dried their hands with a provided paper towel. Women then swabbed the nipple and areola of the breast from which they were planning on collecting milk by wiping a nylon-flocked swab (catalog #552C, COPAN Diagnostics; Murrieta, CA) premoistened with 100 µL Dulbecco's phosphate-buffered saline (DPBS) (Gibco #14190144, Thermo Fisher Scientific, Waltham, MA) in a circular motion for at least 10 sec. The swab head was then broken into a cryovial for storage. Following swabbing, the breast was then washed with soap and warm water for 1 min. After drying the breast with a provided paper towel, participants repeated the swabbing procedure. Women then donned new gloves and while continuing to wear a facemask and gloves, collected up to 30 mL of milk into provided sterile containers. In some cases, milk was aliquoted by the women into cryovials using sterile transfer pipettes. In other cases, the collection container was capped for storage. All samples were stored frozen in the participant’s freezer and shipped to the University of Idaho or to the University of Rochester. When women provided more than one milk sample, pump parts were sterilized using a microwave steam bag (Quick Clean Micro-Steam, Medela). After sterilization, pumps were dried completely and stored in a provided, clean plastic bag until the next sample collection.

**2. Milk samples collected in hospitals**

Sterile milk collection kits (Symphony double pump kit, Medela), breast cleaning, and milk handling instructions similar to those used for at-home collections were provided to participants who were immediately postpartum and had tested positive for COVID-19 per routine on admission to the labor and delivery unit at Brigham and Women’s Hospital (Boston, MA). Milk was self-collected by participants after opening and attaching the sterile pump kit to their hospital grade pump (Symphony, Medela). Samples were stored at -80°C and subsequently shipped on dry ice to the University of Idaho for analysis.

**3. RNA extraction**

Milk was thawed on ice, mixed 1:1 with 2X DNA/RNA Shield (Zymo Research), and incubated for 10 min. For milk analysis, 400 µL of the 1:1 solution or 400 µL of supernatant fluid following centrifugation of the 1:1 solution at 1,600 x g, 15 min at 4°C was used as the input for RNA isolation. For breast swabs, swab heads were immersed in 400 μL 1X DNA/RNA shield (Zymo Research), pulse vortexed for 20 seconds, incubated for 10 min, centrifuged at 500 x g for 1 min at 22°C, and then 400 μL of the liquid aspirated and used as input for RNA isolation. For extraction negative controls, 400 μL 1X DNA/RNA shield was used as the input.

**4. SARS-CoV-2 RT-qPCR assay**

Two methods were employed for the RT-qPCR reactions to detect SARS-CoV-2 in human milk. Both methods used the CDC-designed 2019-nCoV RT-qPCR assay and included three single-plex primer and TaqMan probe sets targeting the N1 and N2 regions of the SARS-CoV-2 nucleocapsid gene as well as the human RNase P gene. Primers and probes were obtained from Integrated DNA Technologies (IDT; Coralville, IA). Genomic SARS-CoV-2 RNA (isolate USA-WA1/2020, catalog # NR-52285) obtained through BEI Resources (Manassas, VA) was used as a standard to ascertain viral genome copy numbers. RT-qPCR method A included use of TaqPath 1-step RT-qPCR master mix, CG (Thermo Fisher Scientific) on an Applied Biosystems Fast 7500 qPCR instrument per the CDC protocol. Briefly, the run mode was set to “standard 7500” and cycling parameters were set to run for 2 min at 25°C; 15 min at 50°C; 2 min at 95°C; followed by 3 sec at 95°C and 30 sec at 55°C for 45 cycles. All samples were run in duplicate as 20 μL reactions, and Ct values ≥40 were interpreted as a negative result. In addition to biological samples, each plate contained a no-template control, inter-assay control (extracted milk with heat-inactivated virus spike-in), and dilution series of SARS-CoV-2 genomic RNA. RT-qPCR method B was similar except it used Bio-Rad Reliance One Step master mix, a Bio-Rad CFX96 instrument (Bio-Rad, Hercules, CA), and primer/probe sets obtained from IDT. Cycling parameters for method B were as follows: 10 min at 50°C and 1 min at 95°C followed by 40 cycles of 3 sec at 95°C and 30 sec at 55°C.

**5. Validation of SARS-CoV-2 RT-qPCR for use with human milk**

To validate the RNA extraction and RT-qPCR for detecting SARS-CoV-2 RNA in human milk, a baseline for the analytical sensitivity/specificity of virus detection and percent recovery of heat-inactivated virus (and viral genomic RNA) present in human milk were determined through a series of experiments. Heat-inactivated SARS-CoV-2 (isolate USA-WA1/2020, catalog #NR-52286), genomic SARS-CoV-2 RNA (isolate USA-WA1/2020, catalog #NR-52285) were obtained through the Biodefense and Emerging Infections Research Resources Repository (BEI Resources, Manassas, VA). Plasmid controls for severe acute respiratory syndrome-associated coronavirus (SARS-CoV; catalog #10006624), Middle East respiratory syndrome coronavirus (MERS-CoV; catalog #10006623), human ribonuclease P protein subunit p30 (RPP30; catalog #10006626) were obtained through IDT (Coralville, IA).

To examine the specificity of the primer/probes used, plasmids targeting SARS-CoV and MERS-CoV were tested in the RT-qPCR assay. No signal was detectable for either plasmid. The effect of RNA isolation method, milk fraction (i.e., whole milk, cell pellet/lipid layer, and supernatant), and storage conditions (i.e., fresh, stored two days at 4°C, and stored seven days at -20°C) on viral detection was evaluated using milk collected from three self-reported healthy women and spiked with known quantities of heat-inactivated virus. Additionally, storage conditions that simulated home sample storage by participants and shipping (i.e., milk stored 24 h in home-style refrigerator freezer, packed in a Milk Stork shipping container with 3 frozen cold packs, stored for 24 h at ~30 °C, thawed on ice for 2 hr, then frozen at -80 °C until extraction) was evaluated. Milk samples were spiked as done previously. Total RNA was isolated from milk samples spiked at a concentration of ~8 genome equivalents/μL heat-inactivated virus (or left unspiked) using silica column (Qiagen Viral RNA mini kit) and magnetic bead-based methods (Zymo Quick-DNA/RNA Viral MagBead kit) (*n* = 108). No false SARS-CoV-2 RNA positives were detected (defined by having at least one duplicate positive for both SARS-CoV-2 N1 and N2 gene targets, both duplicates positive for host RNase P gene target; Ct values <40). Furthermore, the storage/time conditions tested did not have a dramatic impact on the detection of SARS-CoV-2 RNA in milk. Sensitivity of SARS-CoV-2 RNA detection was lowest for whole milk and highest for supernatant, with the supernatant/magnetic bead RNA isolation method having the best performance.

The impact of the milk matrix on percent recovery of virus was also validated by spiking heat-inactivated virus and viral RNA into milk and DPBS prior to and after RNA extraction. Although no difference was found in the percent recovery of virus based on milk fraction or when viral RNA was spiked, Ct values for milk samples spiked with heat-inactivated virus were higher suggesting differences in the efficiency of viral lysis. When Ct values for milk spiked with heat-inactivated virus were normalized to Ct values for DPBS spiked with heat-inactivated virus, only ~3% of virus was lost due to milk matrix effects.

**6. Sample processing for ELISA and microneutralization (MN) assays**

To delipidate milk, samples were thawed on ice and centrifuged at 10,000 x g at 4°C for 10 min. Milk supernatant was collected by piercing the solidified lipid layer with a pipette and carefully withdrawing the soluble phase. The centrifugation was repeated as necessary, and the resulting clear supernatant was used for ELISA assays immediately or aliquoted and frozen for MN assays. If frozen, samples were reclarified by centrifugation as above upon thawing.
